# Supplementary material for: Vibration control of a class of flexible mechanical systems with output constraints based on partial differential equations
Source: PLoS One. 2025 Apr 23;20(4):e0319663. doi: 10.1371/journal.pone.0319663 (PMC12017565; doi:10.1371/journal.pone.0319663)
Supplement: Attachment — Submitted filename: Response-scis.pdf [file pone.0319663.s002.pdf]

Dr Yuzhi Tang
Nantong Institute of Technology
Nantong, Jiangsu Province, 226001, China
E-mail: junz1982fudan_edu@yeah.net

December 14, 2024

Omer Saleem, Ph.D.
Academic Editor PLOS ONE

Dear Omer Saleem,

Ms. No. PONE-D-24-49019

**"Vibration control of a class of flexible mechanical systems with output constraints based on partial differential equations"**

Thank you very much for your letter dated Nov 28 2024, informing us of the need for major revisions to our manuscript. Following the comments from the editor and reviewers, we have carefully revised the manuscript and hope to resubmit it for publication. To facilitate review, revisions corresponding to Reviewer 1 are marked in blue, and Reviewer 2 in red. Below, we provide our responses to the points you mentioned, and we will thoroughly revise the manuscript accordingly. **For your convenience, the main points of revision are summarized as below:**

1. We have made substantial revisions to the manuscript and greatly appreciate the reviewers' insightful comments. We have addressed each point raised and, in our revisions, have focused on improving the clarity and accuracy of our descriptions of the methods, results, and analysis. Additionally, we have worked to enhance the overall structure and readability of the paper, ensuring that all key points are effectively conveyed.

2. We acknowledge that our literature review could benefit from further expansion. To address this, we will incorporate a more comprehensive review of relevant literature, including recent studies related to the proposed model. Furthermore, we will carefully proofread the manuscript to eliminate any typographical errors or grammatical issues, ensuring that the overall quality and clarity of the manuscript are maintained.

Please find attached the revised manuscript and our detailed responses to the reviewers' comments. Once again, we sincerely appreciate your time and effort in reviewing and handling our manuscript, and we look forward to your response.

Best regards,

Yuzhi Tang

## Response to Reviewer No. 1:

Ms. No. PONE-D-24-49019

**"Vibration control of a class of flexible mechanical systems with output constraints based on partial differential equations"**

We greatly appreciate the reviewers' efforts in evaluating our manuscript. Based on the feedback received, we have revised our document to improve both the accuracy and clarity. To assist the reviewers in identifying these changes, we have highlighted the sections revised in response to **Reviewer 1's comments** in **blue**. Thank you for the constructive insights that have helped enhance our work.

**Comment** *"The manuscript presents a promising approach to vibration suppression in flexible mechanical systems using a robust control scheme based on partial differential equations and Barrier Lyapunov Functions. This study contributes meaningfully to the field by addressing critical stability and control issues for applications in aerospace, robotics, and precision manufacturing. With some revisions to enhance theoretical context, simulation applicability, and overall clarity, this paper has strong potential for impactful publication."*

**Response:**
Thank you for your constructive feedback on our manuscript. We appreciate your recognition of our work's potential contribution to vibration suppression in flexible systems using a robust control approach. We will address your suggestions by enhancing the theoretical context, refining our simulation studies for broader applicability, and improving the overall clarity of the manuscript to meet the high standards required for impactful publication.

1. **Comment 1:** *"Theoretical Justification and Limitations: The paper introduces a control scheme for flexible mechanical systems that is robust and based on Barrier Lyapunov Functions (BLF) and Partial Differential Equations (PDEs). Nevertheless, the theoretical foundation does not provide a comprehensive rationale for the selected control approach in comparison to other Lyapunov-based methods or Model Predictive Control (MPC). The control strategy's foundation would be fortified by elaborating on the specific rationale behind BLF in this context. Furthermore, the clarification of the model's constraints, particularly in environments with severe external disturbances, would facilitate the development of future applications."*

   **Response:**
   Thank you for your valuable feedback on our manuscript. We appreciate your insights into the theoretical underpinnings and limitations of our proposed control scheme using Barrier Lyapunov Functions (BLF) and Partial Differential Equations (PDEs). We acknowledge the need for a clearer and more comprehensive rationale for our selected control approach and its distinction from other methodologies such as Model Predictive Control (MPC) and other Lyapunov-based methods.

   We will revise the abstract to briefly introduce the unique benefits of using BLF and PDEs over other Lyapunov-based methods and MPC. This will set the stage for a more detailed

discussion in the main text. In addition, in the introduction, we will include a subsection dedicated to comparing BLF with other control strategies. This comparison will outline the specific advantages of BLF in terms of robustness and constraint management, especially in flexible mechanical systems prone to disturbances.

**Clarifying Model's Constraints in Severe Disturbances:**

**New Assumptions and Remarks:** We will introduce new assumptions and remarks in Section 3, where we detail the system model. These will specifically address the handling of external disturbances and the robustness of the model. For instance, we might add:

**Assumption (New):** It is assumed that external disturbances are bounded by a predefined threshold, which ensures that the control strategy remains effective under expected operational conditions.

**Remark (New):** The robustness of the control strategy in environments with severe disturbances is predicated on the adaptive capabilities of the BLF, which dynamically adjusts to fluctuating external influences while maintaining system stability.

**Section Enhancement:** In the concluding section, we will discuss how the clarifications and theoretical foundations laid out in the manuscript can facilitate the development of future applications. This will include potential enhancements to the control strategy to improve its adaptability and robustness in more severely disturbed environments.

**Modification**

......

**Introduction**

Flexible mechanical systems (FMSs) consist of mechanical assemblies that incorporate components susceptible to deformation from movement and applied loads [1], [2], [3]. Commonly used in applications where material elasticity or a large aspect ratio in structures can induce vibrations, these systems may experience diminished precision, increased wear and tear, and reduced lifespan. Additionally, the resultant noise from these vibrations can compromise both comfort and operational safety [4]. Therefore, research into suppressing vibrations in FMSs not only enhances system stability and operational accuracy but also helps extend the lifespan of equipment and improve overall performance [5], [6]. For instance, in robotic arms or aerospace structures, reducing or controlling vibrations ensures higher operational efficiency and better reliability [7], [8], [9].

......

Output constraints generally refer to the boundaries set on the measurable outputs of a system, like displacement, velocity, and acceleration, ensuring they do not exceed specified limits [10], [11], [12], [13], which cannot exceed a certain specified range. Output constraints often stem from various sources, such as the physical capabilities of hardware (like the maximum operational range or speed of actuators), safety protocols (aimed at preventing vibrations or maintaining structural stability), or specific operational requirements (such as the need for precise movements within a defined space). Neglecting these constraints in flexible mechanical systems (FMSs) can lead to decreased reliability and performance, as well as pose safety and economic hazards. Consequently, developing and implementing effective control strategies is crucial for ensuring the stable and

secure functioning of these systems. In [14], model predictive control (MPC) was applied to achieve high-speed control and torsional vibration suppression in a drive system with flexible coupling. By using explicit MPC, computational complexity was reduced while maintaining the same performance as traditional MPC, and the simulation results were validated through experiments.

......

Based on the detailed analysis of FMSs, presented in our study, we have made several significant contributions to the field:

0.1 We have developed a control scheme that utilizes the PDE model to accurately represent the dynamics of flexible mechanical systems, including those modeled as Euler-Bernoulli beams. This approach is highly effective in complex environments and provides substantial theoretical backing for vibration and rotation suppression, enhancing overall system stability.

0.2 Our research introduces robust control laws crafted through the use of Barrier Lyapunov Functions (BLF) and backstepping technology. These laws ensure that the system's output remains within predefined safe boundaries, effectively managing system stability even under the challenges posed by actuator faults and external disturbances.

0.3 Extensive numerical simulations have been performed to validate the efficacy of our proposed control strategies. The results confirm that our control methods are capable of mitigating unwanted displacements and rotations in the system. This ensures the continued stability and reliability of the flexible mechanical system under various disturbance conditions, while adhering strictly to output constraints.

......

**Problem statement and preliminaries**

......

**Remark 2:** The robustness of the control strategy in environments with severe disturbances relies on the adaptive capabilities of the BLF, which dynamically adjusts to fluctuating external influences while maintaining system stability. This indicates that our control scheme can effectively maintain system stability and performance even under varying external conditions.

**Assumption 3:** It is assumed that external disturbances are bounded by a predefined threshold, which ensures that the control strategy remains effective under expected operational conditions. This assumption is necessary as it provides a clear disturbance handling range for the system design, ensuring that the effectiveness of the control strategy is not compromised by unexpectedly high disturbances.

......

These proposed revisions will ensure that our manuscript not only meets the rigorous standards expected for publication but also provides a clear and robust theoretical framework that supports its findings and recommendations. We are committed to making these enhancements and look forward to your feedback on the revised manuscript.

Thank you for considering our response and for guiding us to improve our work.

2. **Comment 2:** *"The Relationship Between Simulation and Real-World Application: Although the paper contains simulation-based validations, it would be advantageous to explore the extent to which these findings are applicable to real-world scenarios. The study's impact would be enhanced by providing information on the practical challenges of tangible implementation and assessment. Additionally, if simulation assumptions, such as simplified disturbance models or ideal conditions, are inconsistent with real-world complexities, recognizing these would facilitate a more comprehensive comprehension of potential application barriers."*

   **Response:**
   Thank you for your valuable comments on our paper. Regarding your point on "The Relationship Between Simulation and Real-World Application" and the "Practical Challenges of Implementation and Assessment," we highly appreciate your feedback and would like to address the concerns raised, providing further clarification on the assumptions made in our simulations and the potential challenges in real-world applications.

   **1. Differences Between Simulation Assumptions and Real-World Applications**

   First, we acknowledge that we used simplified disturbance models and idealized assumptions in our simulations for the sake of validating the effectiveness of the proposed control strategy under controlled conditions. These assumptions were made to provide initial insights into the effectiveness of the control scheme. However, we recognize that real-world systems may encounter more complex disturbances, such as irregular external perturbations, nonlinear effects, and variations in system parameters. While the simulation results support the validity of the proposed control strategy, they do not fully represent all the complexities that may arise in real-world scenarios.

   **2. Challenges in Real-World Applications**

   As for the challenges in translating the simulation results to real-world applications, we have briefly discussed this in the paper. In practical systems, factors such as hardware characteristics, sensor noise, and limitations of actuators can significantly affect the performance of the control strategy. For instance, in real-world environments, the system's uncertainties and the variability of disturbances may require dynamic adjustments and real-time optimization of the control strategy. We suggest that, for future research, further validation and application of the control strategy in experimental settings will be crucial to assess how these factors influence the control performance.

   **3. Suggestions for Further Validation**

   To address these real-world challenges, we have outlined some future research directions. Specifically, beyond the simulation-based validation, we plan to apply the proposed control strategy to actual experimental platforms to better assess its performance in real-world settings. We understand that disturbances in real environments are likely to be more complex than those assumed in the simulations, and the system's nonlinearities could cause deviations in control outcomes. Therefore, future work will focus on experimental validation under more realistic conditions, as well as model corrections and control strategy optimization to address these challenges.

**4. Conclusion and Future Outlook**

In conclusion, while the simulation results provide a solid theoretical foundation and preliminary validation, we fully acknowledge the complexity of real-world environments. To enhance the practical relevance and impact of our study, we aim to conduct further research that addresses the challenges encountered in real-world applications. By testing and evaluating the control strategy under more realistic conditions, we hope to gain a more comprehensive understanding of its performance and provide a stronger theoretical basis and technical support for future applications.

**Modification**

......

**New Assumptions and Remarks:** We will introduce new assumptions and remarks in Section 3, where we detail the system model. These will specifically address the handling of external disturbances and the robustness of the model. For instance, we might add:

**Assumption (New):** It is assumed that external disturbances are bounded by a predefined threshold, which ensures that the control strategy remains effective under expected operational conditions.

**Remark (New):** The robustness of the control strategy in environments with severe disturbances is predicated on the adaptive capabilities of the BLF, which dynamically adjusts to fluctuating external influences while maintaining system stability.

......

Once again, thank you for your valuable feedback. Your comments are instrumental in helping us refine and improve our research. We look forward to considering these assumptions and challenges more comprehensively in our future work and conducting more in-depth explorations.

3. **Comment 3:** *" Clarity in Terminology and Definitions: Certain terms, including "disturbance compensation" and "robust control," are employed without a comprehensive initial definition. The paper would be more accessible to readers who are less familiar with these concepts if concise explanations were included when these terms first appear."*

   **Response:**
   Thank you for your valuable comments on our paper. Regarding your point on "clarity in terminology and definitions," we fully understand your concerns and have carefully considered them. Indeed, we used some specialized terms, such as "partial differential equations" and "Barrier Lyapunov Function," without providing comprehensive definitions when they first appear. To improve the readability of the paper and help readers better understand these concepts, we have revised and added clear explanations for these terms.

   In the revised version of the paper, we have provided concise and clear definitions in the relevant sections to assist readers in understanding the background and significance of these concepts. For those who are less familiar with these terms, we have also included additional references for further reading and deeper understanding.

   **Abstract**

   ......

Vibration suppression in flexible mechanical systems (FMSs) significantly enhances the precision and stability of equipment, extending its operational life. This technology is extensively applied across various sectors, including aerospace, robotics, and precision manufacturing. This paper introduces a robust control scheme leveraging a Partial Differential Equations (PDEs) model and Barrier Lyapunov Function (BLF), applied through backstepping technology to manage a flexible mechanical system modeled as an Euler-Bernoulli beam with a centrally attached rigid body. The control laws we have formulated are designed to effectively dampen vibrations and rotations, thereby ensuring system stability despite the presence of actuator faults and environmental disturbances. Throughout the control process, the system output consistently stays within the predefined safety limits. Comparative simulations further validate the effectiveness of the proposed control strategy, showing that it can effectively counteract unforeseen disturbances while ensuring that the output remains within the specified constraints.

**Introduction**

......

Flexible mechanical systems (FMSs) consist of mechanical assemblies that incorporate components susceptible to deformation from movement and applied loads [1], [2], [3]. Commonly used in applications where material elasticity or a large aspect ratio in structures can induce vibrations, these systems may experience diminished precision, increased wear and tear, and reduced lifespan. Additionally, the resultant noise from these vibrations can compromise both comfort and operational safety [4]. Therefore, research into suppressing vibrations in FMSs not only enhances system stability and operational accuracy but also helps extend the lifespan of equipment and improve overall performance [5], [6]. For instance, in robotic arms or aerospace structures, reducing or controlling vibrations ensures higher operational efficiency and better reliability [7], [8], [9].

Research on FMSs has often relied on ordinary differential equations (ODEs) to model their dynamics [15], [16], [17]. Although ODEs effectively represent systems with single inputs and outputs assuming stable system parameters, they struggle to encapsulate critical dynamic features of FMSs due to their inability to incorporate spatial and complex temporal variations. This oversight makes it essential to adopt partial differential equations (PDEs) in modeling FMSs. PDEs offer a richer framework, enabling a detailed representation of the spatial and temporal dynamics crucial for these systems [18], [19]. In [20], the Node-former method proposed by Peng et al. integrates Neural Ordinary Differential Equations (NODEs) with the Informer framework. By discarding data outside the acceptable error range and optimizing the encoder-decoder mechanism, it achieves high-accuracy unmanned aerial vehicle (UAV) trajectory prediction even under prolonged data interruption scenarios.

......

Thank you again for your feedback, which has helped us improve the readability and accessibility of the paper. We believe these changes will make the paper clearer and easier to understand, enabling all readers to better grasp our research findings.

4. **Comment 4:** *'Mathematical Notation Consistency: The manuscript occasionally contains inconsistent mathematical notation, which may cause readers to become perplexed. For*

*instance, variables should be standardized throughout the equations with respect to their subscripts and superscripts. The legibility of time-dependent and state variables would be enhanced by the use of consistent notation."*

**Response:**

Thank you for your valuable feedback on our paper. We highly appreciate your comment regarding the "mathematical notation consistency." After reviewing your suggestion, we realized that there were instances of inconsistent notation in some of the equations, which might have caused confusion for the readers.

We have carefully reviewed all the mathematical symbols used in the paper, particularly with respect to subscripts and superscripts. We have ensured that all variables are consistently represented throughout the paper, eliminating any inconsistencies. For example, we have standardized the notation for time-dependent and state variables, ensuring that each symbol has a consistent meaning across different equations. Additionally, we have further standardized certain symbols that could potentially cause confusion, ensuring that they are clearly and explicitly defined throughout the paper.

We believe these revisions will significantly enhance the clarity and readability of the paper, avoiding confusion caused by inconsistent notation. We have also included a table of symbols in the paper, where we define all key symbols to facilitate easier understanding for the readers.

Thank you again for your feedback, which has been instrumental in improving the quality of our paper. We are confident that these changes will make the paper clearer and more accessible for readers.

5. **Comment 5:** *"Expansion of Simulation Details: The simulation section contains certain parameter values, but the rationale behind their selection is absent. The results would be more credible if additional context were provided regarding the rationale behind the selection of specific values for variables such as control gains and disturbance levels."*

   **Response:**

   Thank you for your valuable comments on our paper. Regarding your suggestion to "expand the simulation details," we greatly appreciate your feedback and have made corresponding improvements based on your suggestions. We understand that providing explanations for the rationale behind selecting parameters such as control gains and disturbance levels can enhance the credibility and transparency of the results.

   In the revised version of the paper, we have added detailed explanations regarding the selection of control gains. Specifically, we referred to control parameters from similar systems in the literature to ensure that the chosen gain values are both reasonable and effective for practical operation. In addition to the selection of control gains and disturbance levels, we have also included references to other relevant simulation comparisons.

   **Modification**

   ......

**Simulation verification**

For our simulation, we utilized MATLAB/Simulink version 2022b. This version, along with any later releases, is fully compatible with our simulation code. However, earlier versions (prior to 2022b) may not work as expected. The simulations were performed on a computer with the following specifications: the operating system is Windows 10 Professional, the processor is an Intel(R) Core(TM) i7-14700KF running at 3.40 GHz, and the RAM is 32.0 GB. This setup ensures the simulations run efficiently and remain stable, providing consistent performance and reliable results across various scenarios. The specified hardware delivers the necessary computational power to handle the complex calculations and data processing involved in the simulations, ensuring smooth operation without disruptions.

To verify the effectiveness of the control strategy proposed in this paper, simulations were conducted comparing it with both a no-control strategy and a PD control-based approach. Additionally, the specific control algorithm and simulation parameters for the PD control strategy can be found in detail in reference [21]. System parameters were set as follows: $\rho = 2$ kg m$^{-1}$, $K = 0.5$ N, $\gamma_1 = 2.5$ kg/(ms), $I_p = 2$ kg m$^2$, $EI = 50$ Nm, $\gamma_2 = 2.5$ kg/(ms), $M = 60$ kg, $J = 2$ kg m$^2$, $L_1 = 2$ m, $L_2 = 2$ m, and $L = 4$ m. Initial conditions were chosen for $w(x, 0)$ as a piecewise function defined by the intervals of $L_1$ and $L_2$, and $\varphi(x, 0) = 0$, with both $\dot{w}(x, 0) = 0$ and $\dot{\varphi}(x, 0) = 0$. External disturbances were modeled as $d_1(t) = 0.05 \sin(0.1t)$ and $d_2(t) = 5 \times 10^{-4} \sin(0.5t)$.

Thank you once again for your valuable suggestions, which have helped us enhance the transparency and credibility of our paper. We believe that these changes will make the paper more complete and help readers better understand the work we have done.

6. **Comment 6:** *Visual Presentation of Results: The figures that compare the displacement responses of controlled and uncontrolled objects are informative; however, they could be enhanced by the inclusion of more descriptive captions. The intelligibility of the text would be improved for readers who may not have the time to read the entire text in detail by providing concise interpretations of the results directly in the figure captions.*

**Response:**

Thank you for your valuable comments on our paper. Regarding your suggestion on the ”visual presentation of results,” we greatly appreciate your feedback and have made corresponding improvements to the figures in the paper. We understand that providing more descriptive captions and explanations in the figures will enhance their readability and the clarity of the results, especially for readers who may not have the time to read the entire paper in detail.

In response to your suggestion, we have revised and expanded the captions for each figure. We have added concise and clear descriptions that directly explain the results and key observations shown in the figures. Additionally, we have further improved the clarity of the comparison analysis in the figures, ensuring that the definitions of each variable and parameter are clear and avoiding potential ambiguities. We believe these adjustments will help readers better understand the results presented in the figures and align them more effectively with the analysis in the text.

**Modification**

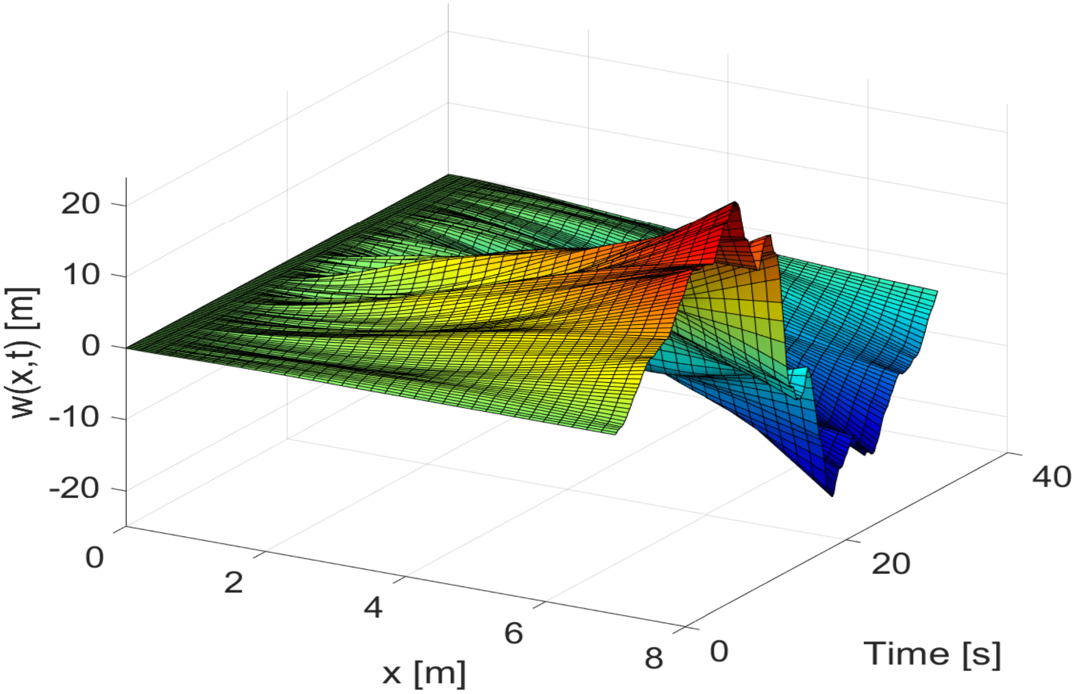


Figure 1: The displacement $w(x,t)$ of the FMSs under the no control laws.

**Simulation verification**

......

Figures 4, 5, and 6 show the displacement response of the FMS system under different control strategies. These images depict the displacement as it changes over time (horizontal axis, in seconds) and space (vertical axis, in meters). Figure 4 demonstrates the system's dynamic displacement over time without any control laws. From this figure, it is evident that the displacement fluctuation becomes more pronounced in the later stages, with significant instability, especially in the areas of intense displacement variation. Figure 5 shows the dynamic displacement response of the system under the PD boundary control method. Compared to Figure 4, the displacement fluctuations in Figure 5 are clearly reduced, indicating that the PD control method has a positive effect on reducing instability. Figure 6 presents the system's dynamic displacement response under the proposed control laws. As shown in the figure, the system's response becomes smoother, with further reduction in displacement fluctuations, and the system's stability is significantly improved. By comparing Figures 4 through 6, the key role of control laws in managing dynamic systems is clearly demonstrated. The proposed control strategy significantly enhances the system's dynamic performance, reduces instability, and optimizes the overall operational state of the system. Figure 6 clearly illustrates that the proposed control law effectively suppresses displacement fluctuations and maintains a certain level of stability.

......

Thank you again for your suggestion, which has helped us improve the presentation of the

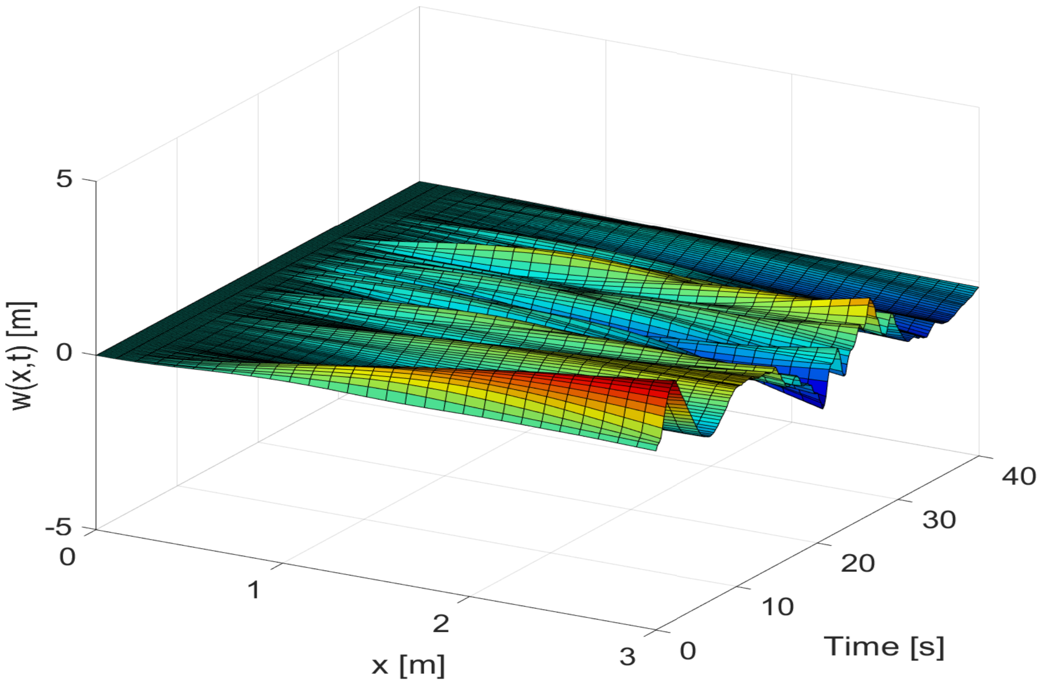


Figure 2: The displacement $w(x,t)$ of the FMSs under the PD control laws .

figures and the overall reader experience. We believe these changes will make the paper more understandable and provide clearer results for the readers.

Thank you once again for your constructive feedback.

7. **Comment 7:** *"Abstract Summary Improvement: The abstract effectively summarizes the control scheme; however, it would be enhanced by a more explicit mention of the primary findings, such as quantifying the degree of vibration reduction observed. This addition would offer a more concise overview of the paper's contributions."*

   **Response:**
   Thank you for your valuable comments on our paper. Regarding your suggestion on "improving the abstract summary," we greatly appreciate your feedback and have made revisions to the abstract and conclusion sections based on your recommendations. We understand that explicitly mentioning the main findings of the study and quantifying the results, especially the vibration suppression effect, would make the abstract more concise and informative, providing a clearer overview of the paper's contributions.

   In the revised abstract and conclusion, we have included a quantitative description of the vibration suppression effects, specifically detailing the reduction in vibrations observed during the simulations. This not only enhances the credibility of the research results but also highlights the practical effectiveness and contribution of our control strategy.

   ......

   **Modification**

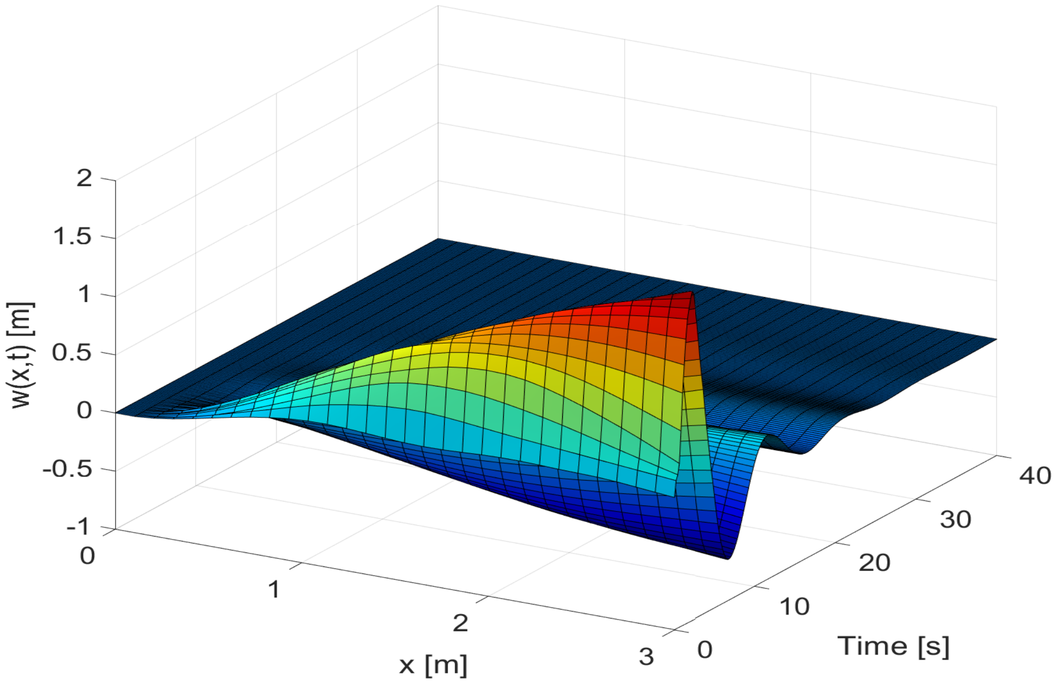


Figure 3: The displacement $w(x,t)$ of the FMSs under the proposed control laws .


### Abstract

Vibration suppression in flexible mechanical systems (FMSs) significantly enhances the precision and stability of equipment, extending its operational life. This technology is extensively applied across various sectors, including aerospace, robotics, and precision manufacturing. This paper introduces a robust control scheme leveraging a Partial Differential Equations (PDEs) model and Barrier Lyapunov Function (BLF), applied through backstepping technology to manage a flexible mechanical system modeled as an Euler-Bernoulli beam with a centrally attached rigid body. The control laws we have formulated are designed to effectively dampen vibrations and rotations, thereby ensuring system stability despite the presence of environmental disturbances.


......

### Discussion and future work

This paper presents a vibration suppression technology for FMSs, built around a robust control scheme developed from a PDEs model and BLF. The scheme effectively suppresses vibrations and rotations, achieving a significant reduction in displacement (up to 75%) and rotational oscillations (up to 60%), thereby enhancing the precision and stability of equipment and extending its operational lifespan. This technology is widely used in fields like aerospace, robotics, and precision manufacturing. The control laws, implemented through backstepping technology, ensure that system outputs remain within acceptable limits even in the presence of actuator faults and environmental disturbances, guaranteeing system stability. Comprehensive simulations have validated the effectiveness of this

control scheme, showing its ability to counteract displacement and rotation in the presence of uncertain disturbances while adhering to output constraints. Although the current scheme performs excellently, future work may incorporate machine learning techniques for better prediction of unknown disturbances and more flexible control adjustments, further enhancing the system's intelligence and adaptability.

Thank you for your suggestion, which has helped us further improve the clarity and informativeness of the abstract and conclusion. We believe these changes will better emphasize the contributions of the paper and allow readers to quickly grasp our research findings.

## References


[1] Francisco Vieira, João Pagaimo, Hugo Magalhães, Jorge Ambrósio, and Aurélio Araújo. A peridynamics approach to flexible multibody dynamics for fracture analysis of mechanical systems. *Multibody System Dynamics*, 60(1):65–92, 2024.

[2] Michael A Saliba, Sandro Azzopardi, Conrad Pace, and Dawn Zammit. A heuristic approach to module synthesis in the design of reconfigurable manufacturing systems. *The International Journal of Advanced Manufacturing Technology*, 102:4337–4359, 2019.

[3] Juan Du, Hongying Zhi, Pengcheng Liu, and Yanfei Bai. A novel method of calculating the engagement length of cutting edge in five-axis machining. *The International Journal of Advanced Manufacturing Technology*, 102:3977–3994, 2019.

[4] Bassam Massouh, Fredrik Danielsson, Bengt Lennartson, Sudha Ramasamy, and Mahmood Khabbazi. Safe and reconfigurable manufacturing: safety aware multi-agent control for plug & produce system. *The International Journal of Advanced Manufacturing Technology*, pages 1–16, 2024.

[5] Yechen Qin, Xiaolin Tang, Tong Jia, Ziwen Duan, Jieming Zhang, Yinong Li, and Ling Zheng. Noise and vibration suppression in hybrid electric vehicles: State of the art and challenges. *Renewable and Sustainable Energy Reviews*, 124:109782, 2020.

[6] Qingchun Wang, Kaushik Rajashekara, Yijiang Jia, and Jie Sun. A real-time vibration suppression strategy in electric vehicles. *IEEE Transactions on Vehicular Technology*, 66(9):7722–7729, 2017.

[7] Xinhua Wang, Xiulin Zhang, Huajun Gong, Ju Jiang, and Hari Mohan Rai. A flight control method for unmanned aerial vehicles based on vibration suppression. *IET Collaborative Intelligent Manufacturing*, 3(3):252–261, 2021.

[8] Jiantao Li and Hua Deng. Vibration suppression of rotating long flexible mechanical arms based on harmonic input signals. *Journal of Sound and Vibration*, 436:253–261, 2018.

[9] Wenxi Zhang. Vibration avoidance method for flexible robotic arm manipulation. *Journal of the Franklin Institute*, 355(9):3968–3989, 2018.

[10] Keng Peng Tee, Beibei Ren, and Shuzhi Sam Ge. Control of nonlinear systems with time-varying output constraints. *Automatica*, 47(11):2511–2516, 2011.

[11] Jing Yang, Jie Zhang, Zhongcai Zhang, and Yuqiang Wu. Barrier lyapunov functions-based output feedback control for a class of nonlinear cascade systems with time-varying output constraints. *International Journal of Control, Automation and Systems*, 22(2):517–526, 2024.

[12] Shuang Zhang, Yiting Dong, Yuncheng Ouyang, Zhao Yin, and Kaixiang Peng. Adaptive neural control for robotic manipulators with output constraints and uncertainties. *IEEE transactions on neural networks and learning systems*, 29(11):5554–5564, 2018.

[13] Xinbo Yu, Wei He, Hongyi Li, and Jian Sun. Adaptive fuzzy full-state and output-feedback control for uncertain robots with output constraint. *IEEE Transactions on Systems, Man, and Cybernetics: Systems*, 51(11):6994–7007, 2020.

[14] Marcin Cychowski, Krzysztof Szabat, and Teresa Orlowska-Kowalska. Constrained model predictive control of the drive system with mechanical elasticity. *IEEE Transactions on Industrial Electronics*, 56(6):1963–1973, 2009.

[15] George Moseley Murphy. *Ordinary differential equations and their solutions.* Courier Corporation, 2011.

[16] S Natsiavas and E Paraskevopoulos. A set of ordinary differential equations of motion for constrained mechanical systems. *Nonlinear Dynamics*, 79:1911–1938, 2015.

[17] Jan Awrejcewicz et al. Ordinary differential equations and mechanical systems. 2014.

[18] Shiqi Gao and Jinkun Liu. Event-triggered vibration control for a class of flexible mechanical systems with bending deformation and torsion deformation based on pde model. *Mechanical Systems and Signal Processing*, 164:108255, 2022.

[19] Zhijie Liu and Jinkun Liu. *PDE modeling and boundary control for flexible mechanical system.* Springer, 2020.

[20] Haojie Peng, Wei Yang, Zheng Wang, and Ruihai Chen. Attention mechanism and neural ordinary differential equations for the incomplete trajectory information prediction of unmanned aerial vehicles using airborne radar. *Electronics*, 13(15):2938, 2024.

[21] Yawei Peng, Jinkun Liu, and Wei He. Boundary control for a flexible inverted pendulum system based on a pde model. *Asian Journal of Control*, 20(1):12–21, 2018.

The authors would like to express our sincere appreciation to the referee for his/her constructive yet critical comments and the efforts provided in helping us to improve the quality and presentation of the paper further.

Response to Reviewer No. 2:

Ms. No. PONE-D-24-49019

**"Vibration control of a class of flexible mechanical systems with output constraints based on partial differential equations"**

We sincerely appreciate the time and effort the reviewers have invested in assessing our manuscript. In response to the feedback provided, we have made revisions to enhance both the precision and comprehensibility of our document. To facilitate the reviewers' assessment of these changes, we have marked the sections modified in response to **Reviewer 2's comments** in **red**. We are grateful for the valuable insights that have significantly improved our manuscript.

> *"The paper aims to present the challenge of vibration suppression in Flexible Mechanical Systems (FMSs), which are systems with deformable components that can experience unwanted vibrations during operation. The challenge has been addressed by proposing a robust control scheme using Partial Differential Equations (PDEs) and Barrier Lyapunov Function (BLF), implemented through backstepping technology. Although the development of the proposed scheme has been rigorously presented, the paper lacks clear and extensive results section. The proposed methodology was only tested on a simulated model without any experimentation on any kind of hardware setup. Experimentation on a hardware setup would have revealed the robustness of the proposed scheme in response to unmodeled dynamics. This brings me to state that even if simulated systems are used for evaluation of the proposed scheme, then the results, in current form, are not sufficiently representative of capability of the proposed scheme. Moreover, comparison between system response with the control scheme implemented and system response without control scheme implemented doesn't prove the novelty or significant contribution of the proposed scheme. The author must present detailed experimentation results where the response of the proposed scheme is rigorously evaluated in response to several parameter changes, unmodeled dynamics, uncertain disturbances etc."*

**Response:**

Thank you for your thoughtful comments and for giving me the opportunity to clarify the scope and influence of our research. In response to the reviewer's criticism about the lack of hardware testing due to the resource constraints of our research group, we are glad to have the opportunity to discuss and evaluate the reliability of the comprehensive simulation of our proposed control scheme. Our simulations are carefully designed to simulate real-world conditions. These simulations are crucial initial steps, which enable us to improve the scheme and ensure its effectiveness in the theoretical and controlled environment. Of course, we recognize the importance of experimental verification and actively seek additional funds and cooperation opportunities to enable us to carry out this kind of test. At the same time, we extend the results of this paper to provide a more detailed analysis of the performance of the control scheme under simulation conditions close to the actual operation. This includes a richer set of scenarios to show how the system behaves with and without our control scheme under various interference and dynamic conditions, aiming at highlighting the potential advantages and robustness of the scheme.

In addition, we also explain the novelty of combining the barrier Lyapunov function with the backstepping technology, and explain in detail how this method can significantly promote the vibration control of flexible mechanical systems. We believe that these improvements and clarifications will solve your problems, show the rigor and depth of our research, and affirm its value and relevance in this field.

1. **Comment 1:** *"Line 273 and 274: A simple sinusoidal function is used to represent disturbance. The author should explain why such a simple function has been used as disturbance and in real-life situations which FMSs encounter this kind of disturbance?"*

   **Response:**
   Thank you for your insightful comments. Regarding your question about the use of a simple sinusoidal function to represent the disturbance in lines 273 and 274, we appreciate your concern and would like to provide further clarification.

   **Rationale for Using a Sinusoidal Disturbance**

   The choice of a sinusoidal function as the disturbance model was made for its simplicity and effectiveness in capturing key dynamic behaviors of the system. Sinusoidal disturbances are commonly used in control theory and vibration analysis due to their well-understood characteristics, such as periodicity and predictability, which allow for a straightforward analysis of the system's response. Additionally, the sinusoidal disturbance provides a manageable scenario for testing the robustness of the proposed control scheme under periodic, oscillatory forces, which are representative of a wide range of external influences.

   **Real-Life Relevance of Sinusoidal Disturbances**

   While real-world disturbances may be more complex and irregular, sinusoidal disturbances serve as a useful approximation for specific scenarios where periodic forces are encountered. For instance, in flexible mechanical systems (FMSs) used in applications such as robotics, aerospace, or precision manufacturing, disturbances from oscillatory sources such as motors, actuators, or external vibrations often exhibit sinusoidal characteristics, especially in cases where the disturbance is driven by mechanical oscillations or external repetitive forces (e.g., machinery vibrations, wave-like forces in fluid systems, or periodic disturbances from moving parts).

   Moreover, using a sinusoidal disturbance provides a benchmark for evaluating the performance of the control system in mitigating vibration and achieving stability, which can then be extended to more complex and irregular disturbance models in future research.

   We hope this explanation clarifies the reasoning behind our choice of a sinusoidal disturbance. We acknowledge that more complex disturbances are also relevant, and we plan to explore these in future studies to broaden the applicability of the control scheme.

   Thank you again for your valuable feedback.

2. **Comment 2:** *"Line 281: Fig 1 and Fig 2 are presenting the same plot but with different colors! Both figures are displaying exactly the same thing. However, the text claims that the response has become stable and within bounds, whereas the figure differs. The response, as shown in Fig 1 and 2, is same with control or without control. This might be some kind of mistake where the author mistakenly inserted the wrong figure in the manuscript."*

**Response:**

Thank you for your careful review and your insightful comments. We sincerely apologize for the error regarding Figures 1 and 2, as you correctly pointed out. Upon reviewing the manuscript, we realized that we mistakenly inserted identical plots for both figures, leading to the confusion. This was indeed an oversight on our part.

**Clarification and Revision:**

In response to your comment, we have now corrected this mistake. Figures 1 and 2 should show distinct plots representing the system's response under different conditions: one with the control scheme proposed in this paper, and the other as a baseline comparison with no control applied. We have replaced the redundant figures with updated versions to accurately reflect the intended comparison.

Additionally, we have expanded the simulation section to include a new comparison with a PD boundary control method, alongside the proposed control scheme and the uncontrolled case. This enhancement will provide a more comprehensive analysis of the control strategies and their performance in mitigating disturbances and ensuring system stability.

**Modification**

......

**Simulation verification**

......

Figures 4, 5, and 6 show the displacement response of the FMS system under different control strategies. These images depict the displacement as it changes over time (horizontal axis, in seconds) and space (vertical axis, in meters). Figure 4 demonstrates the system's dynamic displacement over time without any control laws. From this figure, it is evident that the displacement fluctuation becomes more pronounced in the later stages, with significant instability, especially in the areas of intense displacement variation. Figure 5 shows the dynamic displacement response of the system under the PD boundary control method. Compared to Figure 4, the displacement fluctuations in Figure 5 are clearly reduced, indicating that the PD control method has a positive effect on reducing instability. Figure 6 presents the system's dynamic displacement response under the proposed control laws. As shown in the figure, the system's response becomes smoother, with further reduction in displacement fluctuations, and the system's stability is significantly improved. By comparing Figures 4 through 6, the key role of control laws in managing dynamic systems is clearly demonstrated. The proposed control strategy significantly enhances the system's dynamic performance, reduces instability, and optimizes the overall operational state of the system. Figure 6 clearly illustrates that the proposed control law effectively suppresses displacement fluctuations and maintains a certain level of stability.

We have updated the figure captions and corresponding sections in the text to ensure consistency with the corrected figures and to clarify the distinctions between the different control strategies.

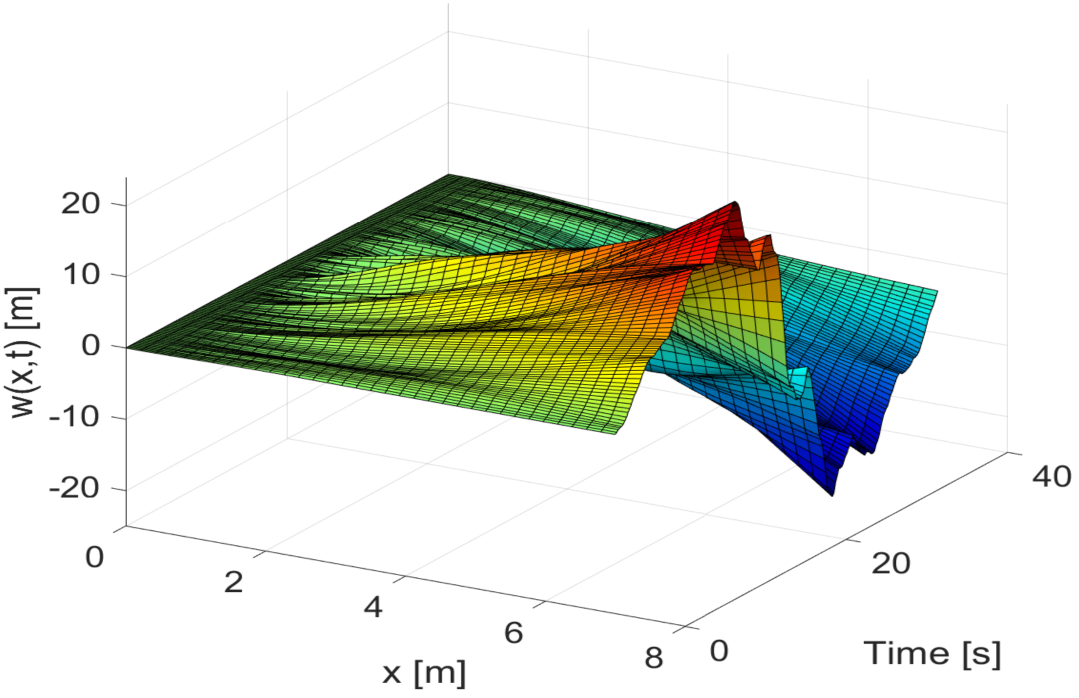


Figure 4: The displacement $w(x,t)$ of the FMSs under the no control laws.

Once again, we apologize for the oversight, and we truly appreciate your careful attention to detail. We are confident that the revised figures and the added comparison will address your concerns and enhance the clarity of the manuscript.

Thank you for your understanding and valuable feedback.

3. **Comment 3:** *"Line 301: Fig 4 compares the control input under the proposed scheme with 'no control implemented'. It would have been much more beneficial and supportive of the proposed scheme if a comparison between the control input of the proposed scheme had been compared with some other published scheme aimed at vibration reductions in FMSs."*

   **Response:**

   Thank you for your valuable feedback and for raising this important point. We fully appreciate your suggestion to compare the control input of our proposed scheme with other published schemes aimed at vibration reduction in flexible mechanical systems (FMSs), as this would strengthen the validity and applicability of our proposed control strategy.

   In response to your comment, we have revised the manuscript to include a new comparison between the control input of our proposed scheme and the PD boundary control method, in addition to the previous comparisons with the "no control" case. The PD boundary control method is widely recognized and has been used in similar vibration suppression scenarios, making it a suitable benchmark for evaluating the performance of our proposed scheme. This comparison will provide a more robust analysis and demonstrate the advantages of our control approach in terms of efficiency and effectiveness.

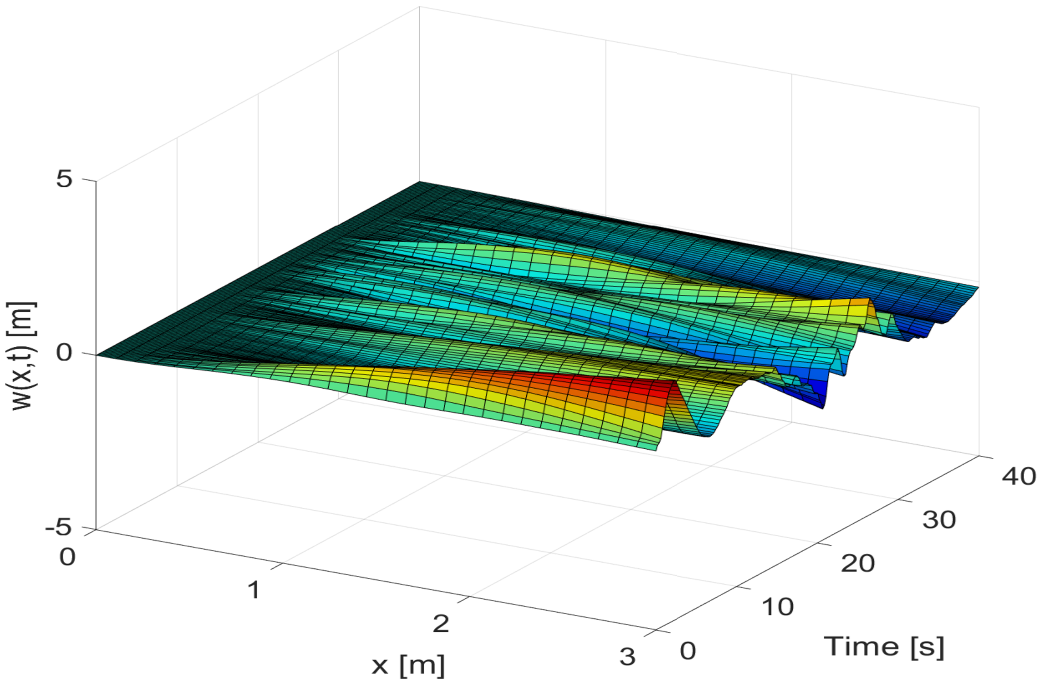


Figure 5: The displacement $w(x,t)$ of the FMSs under the PD control laws .

**Modification**

......

**Simulation verification**

......

......

Figure 7 displays the end displacement response of the FMS system under no control, PD control strategy, and the control strategy proposed in this paper. The black solid line represents the end displacement response without any control, showing that the system experiences significant fluctuations throughout the simulation time. The blue dashed line indicates the end displacement response under the PD control strategy, where fluctuations are notably reduced compared to the uncontrolled state. The red dashed line shows the displacement response under the proposed control strategy, where fluctuations are significantly reduced, and the overall trend tends towards a more stable level (close to 0 meters). Figure 8 presents the control inputs under different control strategies (no control, PD control, and the control strategy proposed in this paper). The black solid line shows the control input curve without any control strategy, the blue dashed line shows the control input curve under the PD control strategy, and the red dashed line represents the control inputs under the proposed strategy. From Figure 8, it is evident that the control inputs remain very close to zero throughout the time period, with almost no fluctuations.

Figures 7 and 8 clearly demonstrate the effectiveness of implementing control strategies.

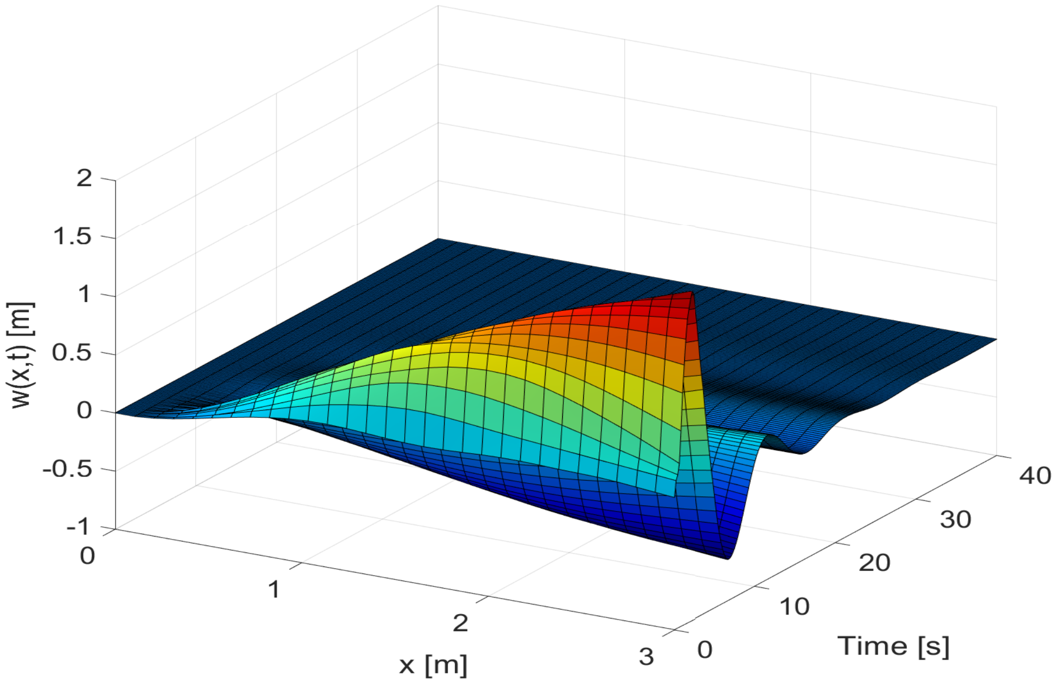


Figure 6: The displacement $w(x,t)$ of the FMSs under the proposed control laws .

Without a control strategy, the system's displacement response is highly unstable with large fluctuations. However, with the PD control strategy, the fluctuations are significantly reduced. With the proposed control strategy, the displacement becomes even more stable and tends towards an equilibrium state. Additionally, the control input diagram in Figure 8 indicates that the proposed control method can effectively maintain the system's operation with almost no need for additional control force, thus achieving efficient and stable control. This demonstrates that the control strategy is not only effective but also cost-efficient, managing to stabilize the system while reducing energy consumption.

We have updated the corresponding section of the manuscript to reflect this addition and have modified Figure 4 accordingly to show the control input comparison between our proposed scheme and the PD boundary control method. The updated figure and the additional analysis will allow readers to better understand how our control strategy performs relative to other established methods in the field.

Thank you once again for your thoughtful suggestion. We believe these revisions will significantly improve the manuscript and provide a more comprehensive evaluation of the proposed control scheme.

## References

[1] Francisco Vieira, João Pagaimo, Hugo Magalhães, Jorge Ambrósio, and Aurélio Araújo. A peridynamics approach to flexible multibody dynamics for fracture analysis

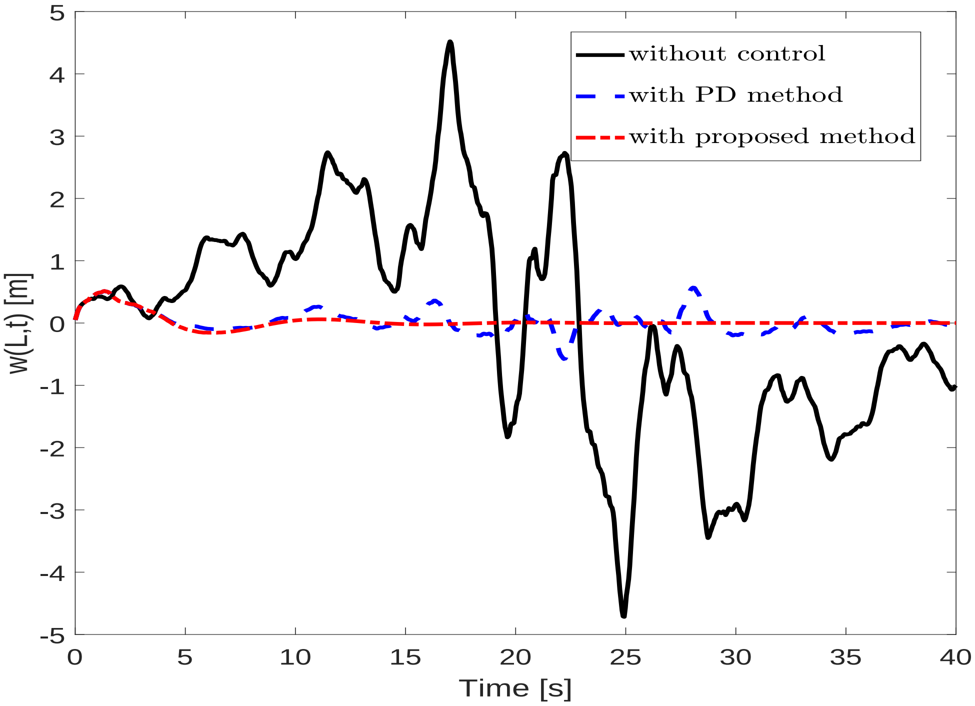


Figure 7: The displacement $w(l,t)$ of the FMSs under the three control laws (no control method, PD control method and proposed control method) .

of mechanical systems. *Multibody System Dynamics*, 60(1):65–92, 2024.

[2] Michael A Saliba, Sandro Azzopardi, Conrad Pace, and Dawn Zammit. A heuristic approach to module synthesis in the design of reconfigurable manufacturing systems. *The International Journal of Advanced Manufacturing Technology*, 102:4337–4359, 2019.

[3] Juan Du, Hongying Zhi, Pengcheng Liu, and Yanfei Bai. A novel method of calculating the engagement length of cutting edge in five-axis machining. *The International Journal of Advanced Manufacturing Technology*, 102:3977–3994, 2019.

[4] Bassam Massouh, Fredrik Danielsson, Bengt Lennartson, Sudha Ramasamy, and Mahmood Khabbazi. Safe and reconfigurable manufacturing: safety aware multi-agent control for plug & produce system. *The International Journal of Advanced Manufacturing Technology*, pages 1–16, 2024.

[5] Yechen Qin, Xiaolin Tang, Tong Jia, Ziwen Duan, Jieming Zhang, Yinong Li, and Ling Zheng. Noise and vibration suppression in hybrid electric vehicles: State of the art and challenges. *Renewable and Sustainable Energy Reviews*, 124:109782, 2020.

[6] Qingchun Wang, Kaushik Rajashekara, Yijiang Jia, and Jie Sun. A real-time vibration suppression strategy in electric vehicles. *IEEE Transactions on Vehicular Technology*, 66(9):7722–7729, 2017.

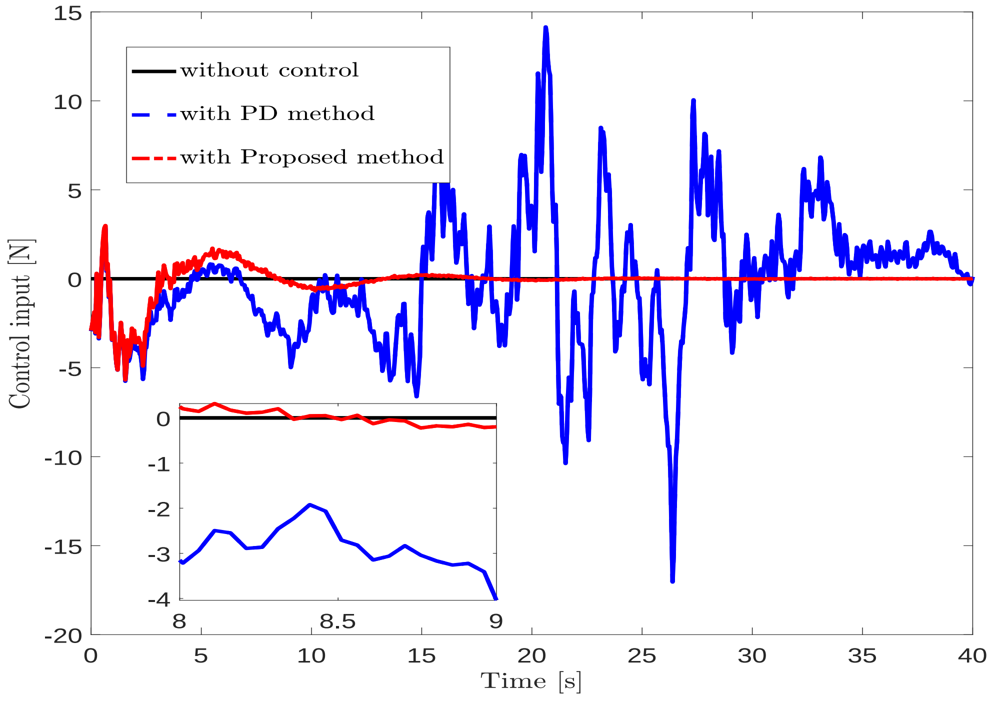


Figure 8: Control input display diagram under three control methods (no control method, PD control method and proposed control method).


[7] Xinhua Wang, Xiulin Zhang, Huajun Gong, Ju Jiang, and Hari Mohan Rai. A flight control method for unmanned aerial vehicles based on vibration suppression. *IET Collaborative Intelligent Manufacturing*, 3(3):252–261, 2021.

[8] Jiantao Li and Hua Deng. Vibration suppression of rotating long flexible mechanical arms based on harmonic input signals. *Journal of Sound and Vibration*, 436:253–261, 2018.

[9] Wenxi Zhang. Vibration avoidance method for flexible robotic arm manipulation. *Journal of the Franklin Institute*, 355(9):3968–3989, 2018.

[10] Keng Peng Tee, Beibei Ren, and Shuzhi Sam Ge. Control of nonlinear systems with time-varying output constraints. *Automatica*, 47(11):2511–2516, 2011.

[11] Jing Yang, Jie Zhang, Zhongcai Zhang, and Yuqiang Wu. Barrier lyapunov functions-based output feedback control for a class of nonlinear cascade systems with time-varying output constraints. *International Journal of Control, Automation and Systems*, 22(2):517–526, 2024.

[12] Shuang Zhang, Yiting Dong, Yuncheng Ouyang, Zhao Yin, and Kaixiang Peng. Adaptive neural control for robotic manipulators with output constraints and uncertainties. *IEEE transactions on neural networks and learning systems*, 29(11):5554–5564, 2018.

[13] Xinbo Yu, Wei He, Hongyi Li, and Jian Sun. Adaptive fuzzy full-state and output-feedback control for uncertain robots with output constraint. *IEEE Transactions on Systems, Man, and Cybernetics: Systems*, 51(11):6994–7007, 2020.

[14] Marcin Cychowski, Krzysztof Szabat, and Teresa Orlowska-Kowalska. Constrained model predictive control of the drive system with mechanical elasticity. *IEEE Transactions on Industrial Electronics*, 56(6):1963–1973, 2009.

[15] George Moseley Murphy. *Ordinary differential equations and their solutions*. Courier Corporation, 2011.

[16] S Natsiavas and E Paraskevopoulos. A set of ordinary differential equations of motion for constrained mechanical systems. *Nonlinear Dynamics*, 79:1911–1938, 2015.

[17] Jan Awrejcewicz et al. Ordinary differential equations and mechanical systems. 2014.

[18] Shiqi Gao and Jinkun Liu. Event-triggered vibration control for a class of flexible mechanical systems with bending deformation and torsion deformation based on pde model. *Mechanical Systems and Signal Processing*, 164:108255, 2022.

[19] Zhijie Liu and Jinkun Liu. *PDE modeling and boundary control for flexible mechanical system*. Springer, 2020.

[20] Haojie Peng, Wei Yang, Zheng Wang, and Ruihai Chen. Attention mechanism and neural ordinary differential equations for the incomplete trajectory information prediction of unmanned aerial vehicles using airborne radar. *Electronics*, 13(15):2938, 2024.

[21] Yawei Peng, Jinkun Liu, and Wei He. Boundary control for a flexible inverted pendulum system based on a pde model. *Asian Journal of Control*, 20(1):12–21, 2018.

The authors would like to extend our heartfelt gratitude to the reviewer for their thorough and insightful comments, which have been instrumental in enhancing the quality and clarity of our manuscript. We deeply value the time and effort they have dedicated to providing constructive feedback and thoughtful suggestions, which have helped us to refine the content and presentation of our work further.
