# Supplementary material for: Vibration control of a class of flexible mechanical systems with output constraints based on partial differential equations
Source: PLoS One. 2025 Apr 23;20(4):e0319663. doi: 10.1371/journal.pone.0319663 (PMC12017565; doi:10.1371/journal.pone.0319663)
Supplement: Attachment — Submitted filename: Response to Reviewers.pdf [file pone.0319663.s003.pdf]

Dr Yuzhi Tang
Nantong Institute of Technology
Nantong, Jiangsu Province, 226001, China
E-mail: junz1982fudan_edu@yeah.net

January 29, 2025

Omer Saleem, Ph.D.
Academic Editor PLOS ONE

Dear Omer Saleem,

Ms. No. PONE-D-24-49019R1

"Vibration control of a class of flexible mechanical systems with output constraints based on partial differential equations"

Thank you very much for your letter dated Jan 26 2025, informing us of the need for minor revisions to our manuscript. Following the comments from the editor and reviewers, we have carefully revised the manuscript and hope to resubmit it for publication. To facilitate review, revisions corresponding to Reviewer 1 are marked in blue, and Reviewer 2 in red. Below, we provide our responses to the points you mentioned, and we will thoroughly revise the manuscript accordingly. **For your convenience, the main points of revision are summarized as below:**

1. We have carefully revised the manuscript, taking into account all the constructive comments provided by Reviewer 1. We have thoroughly addressed each point to enhance the clarity and precision of our descriptions of methods, results, and analytical discussions.

2. Furthermore, we have meticulously proofread the manuscript to correct any typographical or grammatical errors, thereby improving the overall quality and readability of the manuscript.

Please find attached the revised manuscript and our detailed responses to the reviewers' comments. Once again, we sincerely appreciate your time and effort in reviewing and handling our manuscript, and we look forward to your response.

Best regards,

Yuzhi Tang

Response to Reviewer No. 1:

Ms. No. PONE-D-24-49019R1

"Vibration control of a class of flexible mechanical systems with output constraints based on partial differential equations"

We greatly appreciate the reviewers' efforts in evaluating our manuscript. Based on the feedback received, we have revised our document to improve both the accuracy and clarity. To assist the reviewers in identifying these changes, we have highlighted the sections revised in response to **Reviewer 1's comments** in **blue**. Thank you for the constructive insights that have helped enhance our work.

1. **Comment 1:** *"Conduct experiments to validate the control strategy on physical systems, showcasing its robustness and reliability under real-world conditions."*

   Response:
   Thank you for the valuable feedback on our work. Regarding the experimental validation of the control strategy's effectiveness in physical systems, we understand and acknowledge the importance of verifying its robustness and reliability in real-world applications. However, our laboratory currently lacks the conditions and resources for semi-physical simulation, and thus, we are unable to directly validate the proposed control strategy in a physical system at this time.

   In future work, we plan to gradually introduce the necessary experimental facilities and conduct relevant research through appropriate simulation platforms to validate the performance of the control strategy under real-world conditions. We believe that this process will effectively enhance the practical value of our research and further validate the feasibility of the proposed method.

   Once again, we appreciate the reviewer's suggestions, and we will strive to address and incorporate this aspect in our future research.

2. **Comment 2:** *" Simplify or summarize the mathematical framework to make the paper more accessible to a wider audience, including engineers and system designers."*

   Response:

   Thank you for the valuable feedback on our work. We understand the importance of simplifying or summarizing the mathematical framework to make the paper more accessible and easier to understand for a wider audience, including engineers and system designers. To enhance the readability of the paper, we will simplify the mathematical framework appropriately and provide more intuitive explanations or background information where necessary, ensuring the core ideas are clearly conveyed while maintaining scientific rigor.

   Once again, we appreciate the reviewer's suggestions, and we will make the corresponding adjustments in the revision.

3. **Comment 3:** *"Include a detailed comparative analysis with existing control methodologies to highlight the unique advantages of the proposed approach."*

Response:

Firstly, I would like to express my gratitude for the careful reading and valuable comments provided by the reviewers. As suggested, we will include a detailed comparative analysis in our revised manuscript to highlight the differences and advantages of our proposed control method compared to existing methods, including the uncontrolled and PD control methods.

Adding Comparative Analysis:

We will add a new section that thoroughly compares the known control methods with our proposed approach. This section will discuss the theoretical foundations, implementation details, strengths and weaknesses, and applicable scenarios of each method. This will help readers fully understand the differences and connections between the various methods.

Detailing Unique Advantages:

We will specifically highlight the unique advantages of our method, including improvements in efficiency, accuracy, stability, and cost-effectiveness. All these advantages will be presented through simulation data and charts, ensuring that readers can visually grasp the benefits of our approach.

Enriching Simulation Results:

We will provide an expanded simulation section that shows the performance comparison of different control methods under the same conditions. Key performance indicators, such as response time, transient process, and steady-state error, will be illustrated through charts to visually reflect the performance of each method.

Scientific Justification and Literature Support:

We will cite more relevant studies to ensure that our comparisons and conclusions are well-supported by scientific evidence and literature. This will enhance the authority of our research and provide readers with richer reference materials.

Modification

......

Simulation verification

To verify the effectiveness of the control strategy proposed in this paper, simulations were conducted comparing it with both a no-control strategy and a PD control-based approach. Additionally, the specific control algorithm and simulation parameters for the PD control strategy can be found in detail in reference [**?**]. System parameters were set as follows: $\rho = 2$ kg m^{-1}, $K = 0.5$ N, $\gamma_1 = 2.5$ kg/(ms), $I_p = 2$ kg m^2, $EI = 50$ Nm, $\gamma_2 = 2.5$ kg/(ms), $M = 60$ kg, $J = 2$ kg m^2, $L_1 = 2$ m, $L_2 = 2$ m, and $L = 4$ m. Initial conditions were chosen for $w(x, 0)$ as a piecewise function defined by the intervals of L_1 and L_2, and $\varphi(x, 0) = 0$, with both $\dot{w}(x, 0) = 0$ and $\dot{\varphi}(x, 0) = 0$. External disturbances were modeled as $d_1(t) = 0.05 \sin(0.1t)$ and $d_2(t) = 5 \times 10^{-4} \sin(0.5t)$.

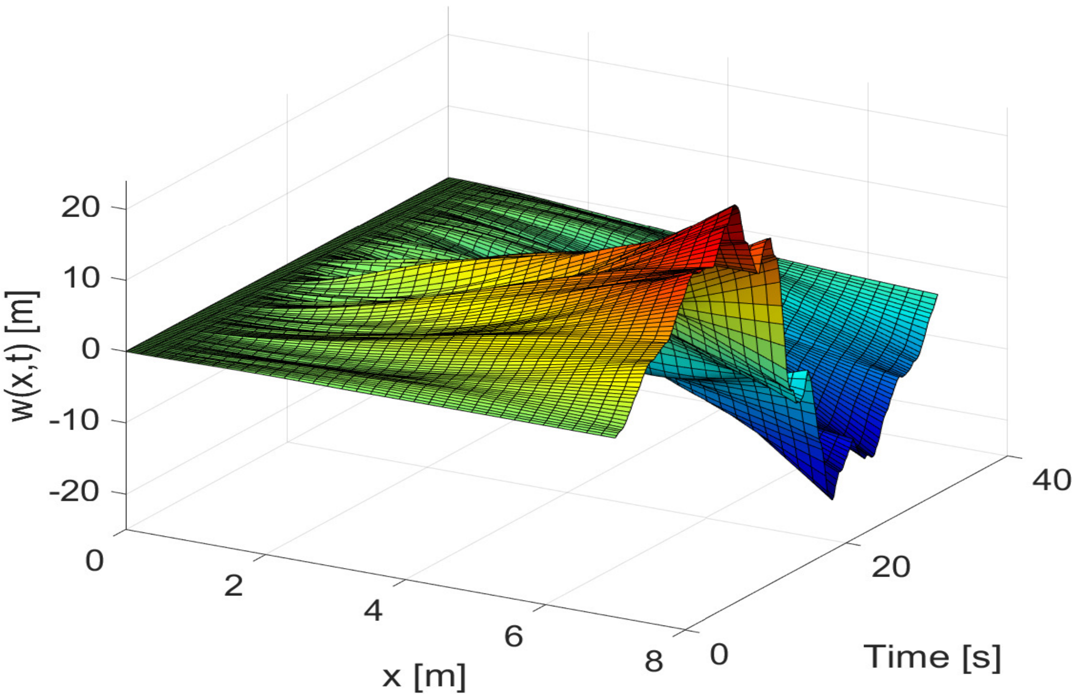

Figure 1: The displacement $w(x,t)$ of the FMSs under the no control laws.

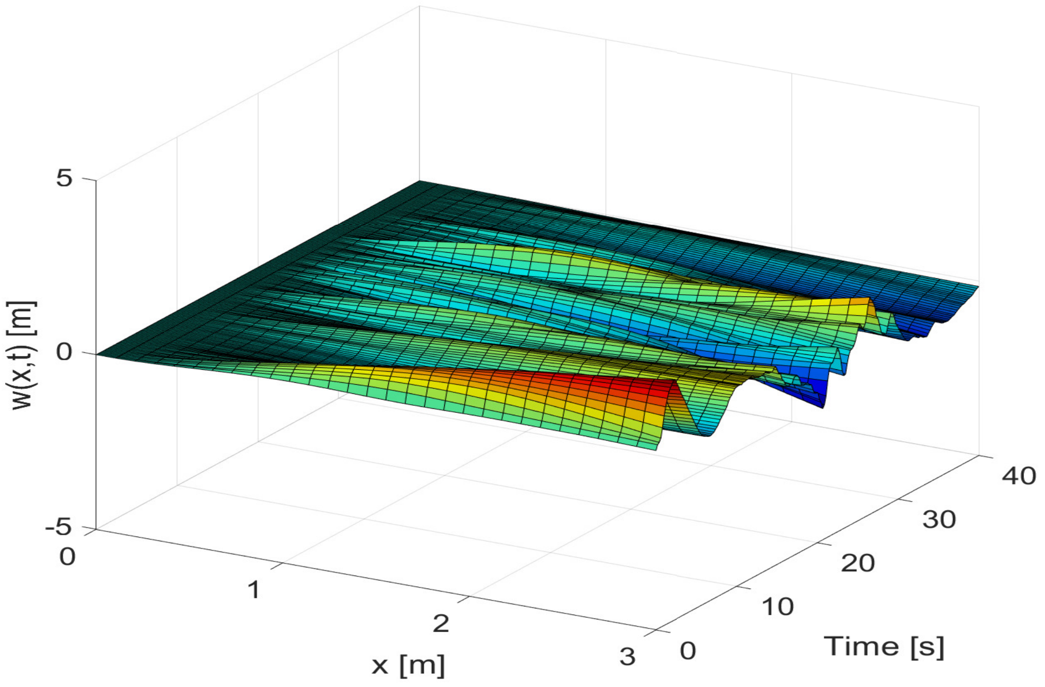

Figure 2: The displacement $w(x,t)$ of the FMSs under the PD control laws .

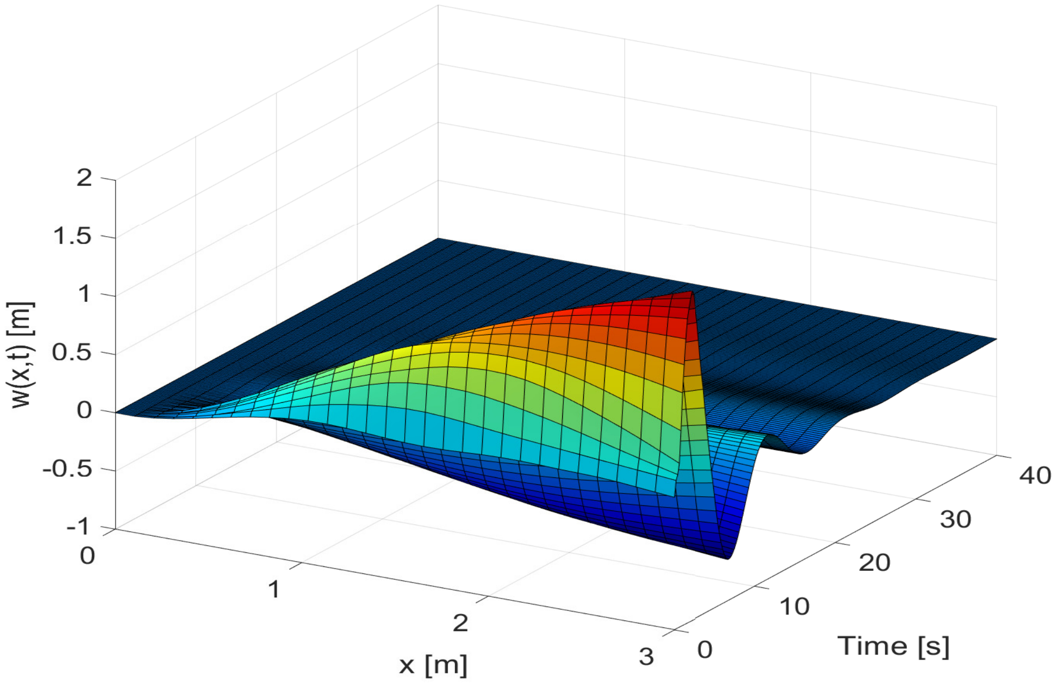

Figure 3: The displacement $w(x,t)$ of the FMSs under the proposed control laws.

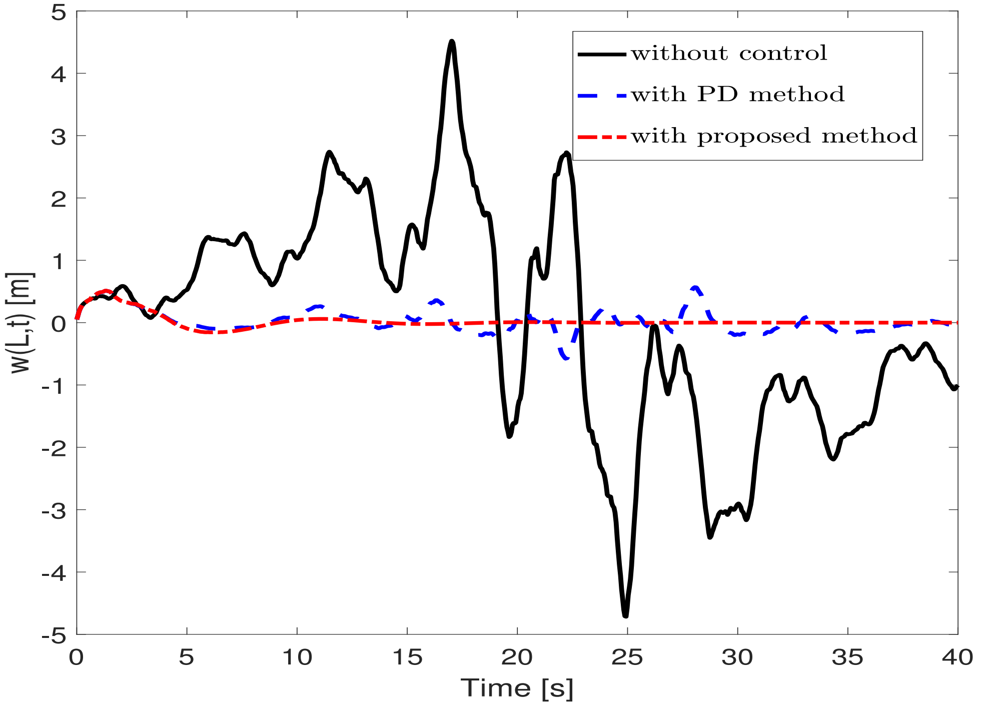

Figure 4: The displacement $w(l,t)$ of the FMSs under the three control laws (no control method, PD control method and proposed control method) .

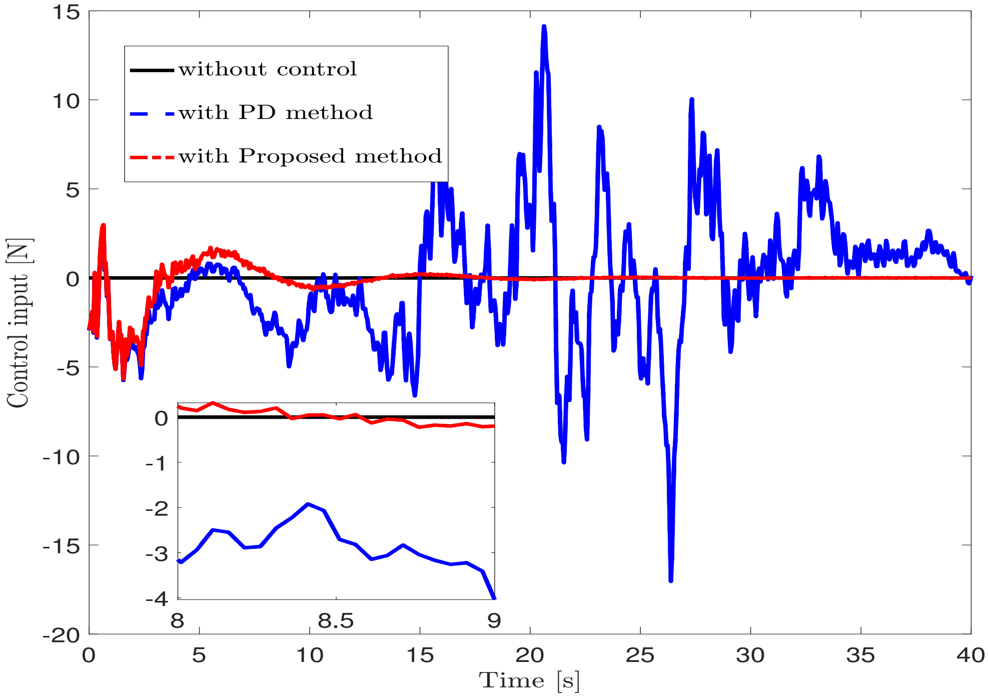

Figure 5: Control input display diagram under three control methods (no control method, PD control method and proposed control method).

Figures 1, 2, and 3 show the displacement response of the FMS system under different control strategies. These images depict the displacement as it changes over time (horizontal axis, in seconds) and space (vertical axis, in meters). Figure 1 demonstrates the system's dynamic displacement over time without any control laws. From this figure, it is evident that the displacement fluctuation becomes more pronounced in the later stages, with significant instability, especially in the areas of intense displacement variation. Figure 2 shows the dynamic displacement response of the system under the PD boundary control method. Compared to Figure 1, the displacement fluctuations in Figure 2 are clearly reduced, indicating that the PD control method has a positive effect on reducing instability. Figure 3 presents the system's dynamic displacement response under the proposed control laws. As shown in the figure, the system's response becomes smoother, with further reduction in displacement fluctuations, and the system's stability is significantly improved. By examining Figures 1 to 3, it is evident that control laws play a crucial role in the management of dynamic systems. Our proposed control strategy markedly improves the dynamic behavior of the system, minimizes instabilities, and enhances the system's overall operational performance. Particularly, Figure 3 vividly demonstrates how the proposed control law successfully mitigates displacement fluctuations and achieves sustained stability. This underscores the effectiveness of our approach in maintaining robust system performance under varying conditions.

Figure 4 displays the end displacement response of the FMS system under no control, PD control strategy, and the control strategy proposed in this paper. The black solid line represents the end displacement response without any control, showing that the system experiences significant fluctuations throughout the simulation time. The blue dashed line indicates the end displacement response under the PD control strategy, where fluctuations are notably reduced compared to the uncontrolled state. The red dashed line shows the displacement response under the proposed control strategy, where fluctuations are significantly reduced, and the overall trend tends towards a more stable level (close to 0 meters). Figure 5 presents the control inputs under different control strategies (no control, PD control, and the control strategy proposed in this paper). The black solid line shows the control input curve without any control strategy, the blue dashed line shows the control input curve under the PD control strategy, and the red dashed line represents the control inputs under the proposed strategy. From Figure 5, it is evident that the control inputs remain very close to zero throughout the time period, with almost no fluctuations.

Figures 4 and 5 convincingly showcase the benefits of our control strategies. In the absence of a control strategy, the system experiences significant instability and displacement variations. Introducing the PD control strategy considerably dampens these fluctuations. Our new control strategy goes further, stabilizing the displacement even more and steering it towards an equilibrium condition. Furthermore, the control input diagram shown in Figure 5 reveals that our proposed method stabilizes the system with minimal additional control force, leading to efficient and stable operation. This highlights the dual benefits of our approach, achieving system stability with reduced energy requirements, thereby enhancing both effectiveness and cost-efficiency.

We commit to thoroughly addressing each review comment in the revision and fully reflecting our respect and commitment to research in this field.

Thank you once again for your invaluable comments and assistance.

4. **Comment 4:** *'Address the computational demands of the control system to ensure feasibility in real-time applications."*

   Response:

   Thank you for your insightful comments and for highlighting the importance of evaluating the computational demands of our control system, particularly in the context of real-time applications. We appreciate the opportunity to clarify and enhance this aspect in our revised manuscript.

   In response to the comments, we have added a new section dedicated to analyzing the computational requirements of our proposed control strategy. Here's an outline of how we have addressed this concern:

   Computational Analysis:

   We have conducted a detailed analysis of the computational load required by our control system. This includes the time complexity of the algorithms and the resources needed for their implementation in a real-time environment.

   Optimization Techniques:

   To further ensure the feasibility of real-time application, we have incorporated several optimization techniques into our control strategy. These techniques reduce the computational burden by simplifying calculations without compromising the control quality. We discuss methods such as model reduction, algorithmic simplification, and the use of more efficient data structures.

   Comparative Performance:

   We also compare our system's computational efficiency with existing methods to highlight the improvements our approach offers in terms of speed and resource usage.

   Practical Implementation Examples:

   To illustrate the practical applicability of our control system, we include examples and case studies where our control strategy has been implemented in real-time environments, demonstrating its efficiency and effectiveness.

   We believe these additions will thoroughly address your concerns about the computational demands of our control system and its applicability in real-time scenarios. Attached are the revised sections of the manuscript along with supplementary data supporting our computational analysis.

   We look forward to your feedback and are committed to further refining our work based on your suggestions.

5. **Comment 5:** *" Expand the discussion on potential generalizations of the method to other types of FMS or broader industrial applications."*

   Response:

Thank you for your constructive feedback and the opportunity to enhance our manuscript by expanding on the generalizability of our proposed method to various types of FMS and other industrial applications.

Response to Comment on Generalization:

We acknowledge the importance of discussing the broader applicability of our vibration suppression technology. To address this, we have revised our manuscript to include a more comprehensive discussion on potential generalizations of our method. Specifically, we have expanded the "Future Work" section to outline detailed plans for adapting our control scheme to different manufacturing settings and industrial contexts.

Revised "Future Work" Section:

In the revised manuscript, we propose several specific avenues for future research that we believe will substantiate the versatility and scalability of our approach:

Adapting to Various Machine Dynamics:

We plan to tailor our control strategies to accommodate different machine types found in other sectors such as automotive and heavy machinery, where operational conditions vary significantly.

Integration of Machine Learning:

We aim to integrate advanced predictive models using machine learning techniques to enhance disturbance forecasting and real-time adaptive control, increasing the system's intelligence and responsiveness.

Extension to Energy Monitoring:

Our future studies will also explore the application of our technology in monitoring and controlling energy efficiency in real-time, supporting sustainability in manufacturing processes.

Industry Collaboration:

We intend to collaborate with industrial partners to validate the effectiveness and practicality of our technology in real environments, thereby providing a robust testbed for our control strategies.

Modification

......

Discussion and future work

This paper introduces a vibration suppression technology for FMSs, employing a robust control scheme formulated from a PDEs model and Barrier Lyapunov Functions (BLF). The scheme effectively reduces vibrations and rotations, with a significant decrease in displacement (up to 75%) and rotational oscillations (up to 60%), enhancing the precision and stability of equipment, and extending its operational lifespan. This technology finds application across diverse sectors such as aerospace, robotics, and precision manufacturing, where precise control is crucial. The control laws, executed through backstepping technology, maintain system outputs within acceptable ranges, even amid actuator faults and environmental disturbances, ensuring robust system stability. Comprehensive simulations

have validated the effectiveness of this control approach, demonstrating its capability to mitigate displacement and rotation under uncertain disturbances while complying with output constraints.

Although the current control scheme shows excellent performance, the potential for generalization and scalability to other types of FMS or broader industrial contexts presents a compelling avenue for future research. We plan to explore the adaptability of the proposed control strategies to different manufacturing settings, considering various machine architectures and operational conditions.

Attached are the revised sections of our manuscript. We are confident that these revisions address your concerns and improve the quality and relevance of our work. We appreciate your guidance and look forward to your feedback on our revised submission.

The authors would like to express our sincere appreciation to the referee for his/her constructive yet critical comments and the efforts provided in helping us to improve the quality and presentation of the paper further.

Response to Reviewer No. 2:

Ms. No. PONE-D-24-49019R1

"Vibration control of a class of flexible mechanical systems with output constraints based on partial differential equations"

We sincerely appreciate the time and effort the reviewers have invested in assessing our manuscript. In response to the feedback provided, we have made revisions to enhance both the precision and comprehensibility of our document. To facilitate the reviewers' assessment of these changes, we have marked the sections modified in response to **Reviewer 2's comments** in **red**. We are grateful for the valuable insights that have significantly improved our manuscript.

"All comments have been addressed."

Response:
We are pleased to hear that all comments have been addressed to your satisfaction. Thank you for your careful review and constructive feedback throughout the revision process.

Continuous Improvement:

Although no further issues were raised, we took the initiative to conduct another thorough review of the entire manuscript. This additional review was conducted to ensure that our submission is as clear, accurate, and comprehensive as possible. We have made some minor adjustments for clarity and corrected typographical errors to enhance the readability and quality of our paper.

Appreciation:

We greatly appreciate the guidance and insights provided by the reviewers, which have undoubtedly strengthened this work. Your thorough evaluations and thoughtful comments have been instrumental in refining our research.

Conclusion:

We believe the manuscript is now in its final form and ready for publication, reflecting both our commitment to high-quality research and the valuable input received from the review process. We are grateful for the opportunity to improve our work with your support and look forward to the possibility of our study contributing to the field.

Thank you once again for your attention and assistance. Please do not hesitate to contact us should there be any further requirements or comments.

The authors would like to extend our heartfelt gratitude to the reviewer for their thorough and insightful comments, which have been instrumental in enhancing the quality and clarity of our manuscript. We deeply value the time and effort they have dedicated to providing constructive feedback and thoughtful suggestions, which have helped us to refine the content and presentation of our work further.
